# Supplementary material for: A Global Screen for Assembly State Changes of the Mitotic Proteome by SEC-SWATH-MS
Source: Cell Syst. 2020 Feb 26;10(2):133–155.e6. doi: 10.1016/j.cels.2020.01.001 (PMC7042714; doi:10.1016/j.cels.2020.01.001)
Supplement: Data S1. SEC-SWATH-MS Protein Chromatograms, Related to Figure 1 [file mmc6.zip › SECchrom_Q9BV86_NTM1A_HUMAN_NTMT1_C9orf32_METTL11A_NRMT_NRM.pdf]

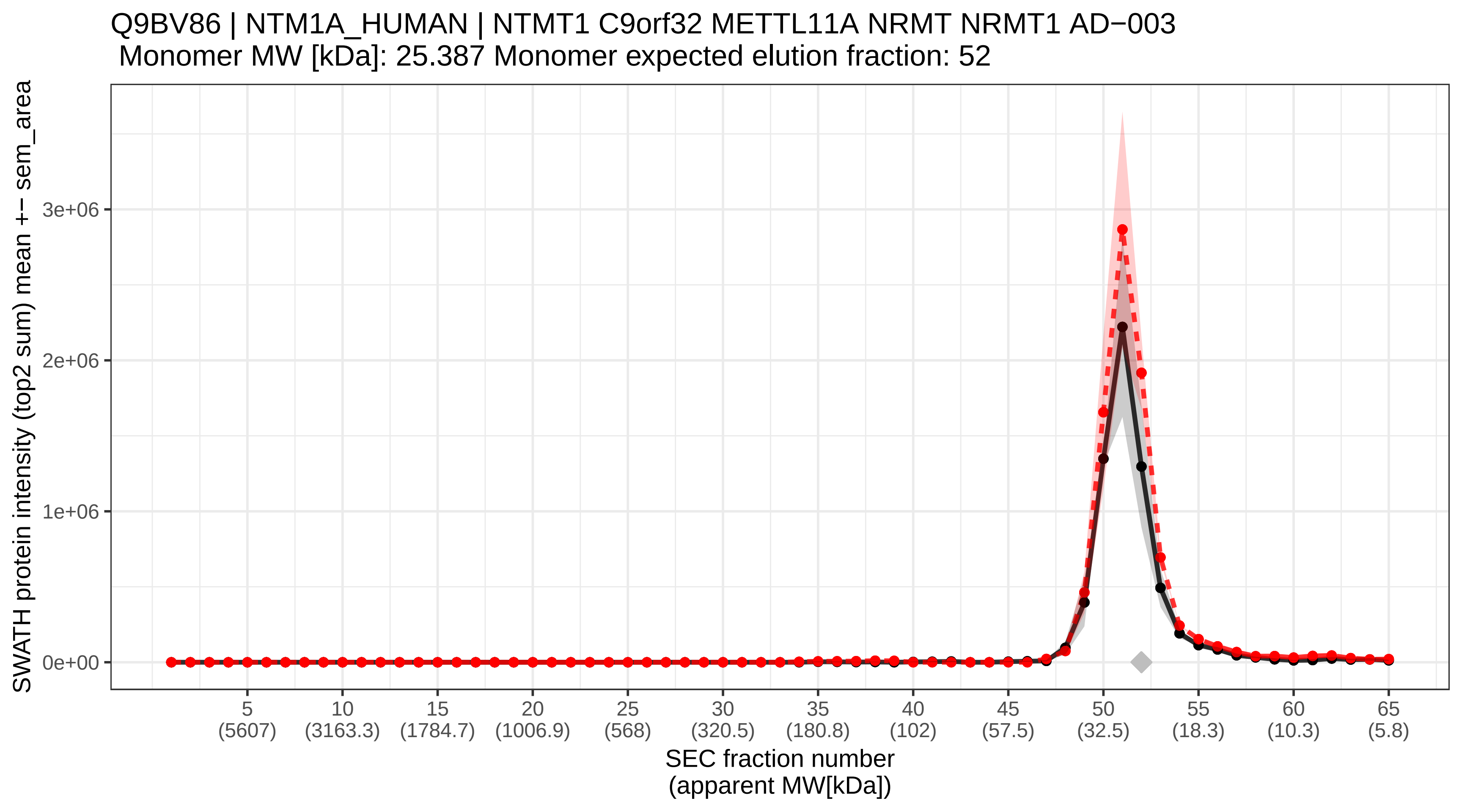

Q9BV86 | NTM1A_HUMAN | NTMT1 C9orf32 METTL11A NRMT NRMT1 AD−003
Monomer MW [kDa]: 25.387 Monomer expected elution fraction: 52
SWATH protein intensity (top2 sum) mean +− sem_area
3e+06
2e+06
1e+06
0e+00
5 (5607)
10 (3163.3)
15 (1784.7)
20 (1006.9)
25 (568)
30 (320.5)
35 (180.8)
40 (102)
45 (57.5)
50 (32.5)
55 (18.3)
60 (10.3)
65 (5.8)
SEC fraction number
(apparent MW[kDa])
